# Using Social Media to Communicate Sustainable Preventive Measures and Curtail Misinformation

**DOI:** 10.3389/fpsyg.2020.568324

**Published:** 2020-10-16

**Authors:** Michael K. Hauer, Suruchi Sood

**Affiliations:** Dornsife School of Public Health, Drexel University, Philadelphia, PA, United States

**Keywords:** social media, health communication, misinformation, COVID-19, preventive measures

## Abstract

Effective crisis and risk communication strategies are crucial to promote preventive measures, particularly during times of emergency such as the global SARS-CoV-2 (COVID-19) pandemic. With its global reach, social media is a key source of news and information about COVID-19. However, the abundance of misinformation about personal protective measures that people post on social media, makes it imperative to develop a deeper understanding of effective messaging strategies. Improving the quality of information and strategy with which it is disseminated through social media is crucial to minimizing anxiety, panic and improving the adoption of sustainable preventive measures in addition to curtailing misinformation. Understanding the components of effective health communication strategies allows us to glean common methods to address misinformation which in turn lead to people adopting the appropriate preventive measures. The purpose of this article is to understand how effective social media communication strategies can be crafted to promote sustainable preventive measures and curtail wide-spread misinformation. Health organizations as well as communications organizations have made available information for effective social media messaging and more importantly serve as a gateway to other resources. We review their recommendations to identify common social media communication elements on the adoption of sustainable preventive measures and effective strategies for curtailing misinformation. We further review social media messaging during the Ebola and Zika outbreaks to evaluate the success of social media strategies and draw from lessons learned. We then create a set of best practices for developing and disseminating social media messaging regarding COVID-19.

## Introduction

In less than 6 months SARS-CoV-2 (COVID-19) has grown from a localized outbreak in Wuhan, China to a global pandemic (Johns Hopkins University and Medicine, 2020). The exponential spread has left countries and health officials scrambling to contain the virus and protect their citizens. Currently there is no vaccine for COVID-19 and with an incubation period of up to 14 days, the virus has proven extremely difficult to contain (Lauer et al., 2020). The rapid rate at which the scientific community is learning about COVID-19 and personal protective measures have created a need for regular, easily accessible, up-to-date, and accurate information. The dramatic changes in our daily lives have had an enormous impact on our behaviors including where we turn for news. As the scientific community learns more, there is a dire need to be able to disseminate information immediately; social media provides a platform that can facilitate this.

On March 27th, António Guterres, the Secretary General of the World Health Organization, posted on Twitter, “Our common enemy is #COVID-19, but our enemy is also an ‘infodemic’ of misinformation. To overcome the #coronavirus, we need to urgently promote facts and science” (United Nations, 2020). Developing effective social media strategies that provide accurate information about COVID-19 preventive measures is of the utmost importance.

In 2018, Guardian columnist Natalie Nougayrède said “The use of [misinformation] is ancient, but never before has there been the technology to so effectively disseminate it” (Nougayrede, 2018). Curtailing misinformation and developing effective social media messaging that increase the adoption of preventive measures is of the utmost importance. In the face of a pandemic, developing effective risk and emergency social media strategies is also crucial to counteract misinformation. By providing up to date and accurate information, promoting preventative messages, bringing together communities of individuals and experts along with celebrities’ role modeling healthy behaviors, social media has the potential to be a powerful tool that could increase the adoption of sustainable preventive measures. This article outlines seven best practices for developing social media messaging about COVID-19.

## Misinformation and Information Processing

How people process information is rooted in the work of Baruch Spinoza and René Descartes. Descartes argued that people typically screen out and process misinformation shortly after being exposed to it. Spinoza countered Descartes’ by espousing that people accept all information they are exposed to as truth and verify it or reject it in a subsequent process (University of Pennsylvania Annenberg School of Communication, 2017). While much of the work on information processing is attributable to Spinoza and Descartes, it has continued to be a focus of research psychologists. Events such as 9/11, elections, Ebola, Zika, and most recently COVID-19 has led to several studies that explore why people accept or reject misinformation or conspiracy theories. Particular emphasis has been placed on whether people are predisposed to reject official accounts of major issues (conspiracy ideation) or whether they have specific beliefs about specific theories (conspiracy beliefs) (Klofstad et al., 2019). Several studies have found that conspiracy ideation is a better predictor of whether or not a person will accept or reject misinformation (Klofstad et al., 2019; Business of Apps, 2020; Uscinski et al., 2020).

Lantian et al. (2017) identified several characteristics of a person that is likely to believe in conspiracy theories including an openness to experience, low agreeability, distrust, and Machiavellianism (Grohol, 2018). A study by Uscinski et al. (2020) that focused specifically on conspiracy theories and misinformation regarding COVID-19 supported the findings of Lantian et al. (2017) and also mentioned ideological motives and the politicization of major events such as COVID-19 as potential reasons for why a person might believe a conspiracy theory (Ireton and Posetti, 2018; Grohol, 2018). One finding of the study was that 29% of respondents agreed that the severity of COVID-19 had been exaggerated to damage the reputation of President Trump. Understanding the underlying tendencies and reasons that lead a person to accept or reject misinformation is crucial to understand when developing effective, accurate, and expedient social media strategies during the COVID-19 pandemic.

## Social Media During COVID-19

Social media use as a source of information and entertainment has grown exponentially over the last decade. The top five platforms are Facebook with 2.26 billion users, YouTube with 1.9 billion, WeChat with approximately 1 billion, Instagram with 1 billion users (Ortiz-Ospina, 2019), and TikTok with 500 million users (Carey et al., 2020; Statista, 2020). Users are often active on multiple platforms, so it is difficult to provide an estimate on exposure to specific information. The rapid global spread of COVID-19 has altered where people turn for news and updates about the virus. A recent report that analyzed media behaviors during the pandemic reported that 40% of people visit social media more for news than they did before COVID-19 (Havas Media Group, 2020). Despite consuming more news on social media, a recent survey by Axios-Harris reported that out of 13 sources of news that were identified, respondents answered that they trusted news or information posted on social media the least (Harris Insights and Analytics, 2020).

While misinformation is widespread on social media, it is important to note the positive role that social media can play if used to disseminate clear and accurate information about COVID-19. Dr. Jemilah Mahmood, Undersecretary General for Partnerships at the International Federation of Red Cross and Red Crescent Societies, said of social media during a crisis “by engaging with social media as standard practice in the aftermath of an emergency, we can understand what people are worried about; we can see news they are sharing; and we can respond decisively, accurately, and collaboratively” (International Federation of Red Cross and Red Crescent Societies, 2017; Posetti and Matthews, 2018). Social media gives health organizations the ability to disseminate information and update the public almost instantly. It also provides an opportunity for health organizations to gain a deeper understanding of misconceptions about COVID-19 and information that the public wants. Understanding how to craft and frame effective social media messaging in an engaging and approachable way is necessary to capture the attention of the public and curtail misinformation.

## Methods

We conducted five reviews to identify a set of best practices for effective social media messaging to promote sustainable protective measures and curtail misinformation. Successful and unsuccessful strategies were identified to help inform the best practices detailed in the “Discussion” section.

**FIGURE 1:**
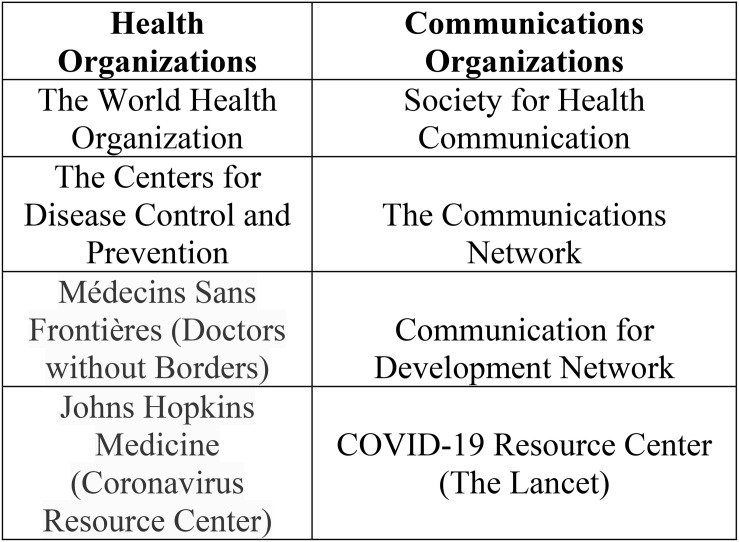
Health and communications organizations.

First, we reviewed documents from the four health organizations in Figure 1 that contained recommendations for effective social media messaging. These health organizations were selected for review because of their large social media following and the integral role they have played in disseminating accurate and up-to-date information about COVID-19 preventive measures. Second, we reviewed suggestions for effective social media messaging made by the four communications organizations in Figure 1. These communications organizations were selected because they served a dual purpose by providing specific recommendations and providing resources for our third review while many other communications organizations only provided recommendations or shared resources. In our third review we reviewed information about effective social media messaging from the additional resources that were provided by the communications organizations. These communications organizations provided links to articles with sets of recommendations, or specific examples of social media messages from the New York Times, Office of Disease Prevention and Health Promotion, PolitiFact’s, and Vox and other news outlets and government agencies that were not part of the first two reviews and may not have been identified otherwise. These websites were explored in depth until we reached a saturation point where we believed all of the information regarding COVID-19 social messaging had been identified. Fourth, we examined social media use during the Ebola and Zika outbreaks by referencing several peer-reviewed articles to understand how social media messages were crafted during the Zika and Ebola outbreaks. Fifth, we reviewed social media strategies from countries such as Vietnam that are considered success stories in controlling COVID-19.

We defined a best practice as a recommendation made by multiple health and/or communications organizations or a strategy that was used in countries that have low transmission rates and deaths. A suggestion that is made for crafting social media messaging by one organization is not sufficient regardless of the organization’s prestige. Most organizations have their own terminology so it is critical to understand synergies, contradictions, and contextual factors that may affect the development of their social media guidelines or practices in their respective recommendations. These best practices can also build upon successful strategies utilized during the Ebola and Zika outbreaks.

## Discussion

From these five reviews, seven best practices were identified to inform effective social media messaging to curtail misinformation and promote sustainable preventive measures during COVID-19.

### Framing Risk to Promote Preventive Measures and Reduce Panic

Social media messages that effectively frame risk have the potential to reduce panic and increase adoption of preventive measures by conveying what behaviors and decisions put a person at increased risk of developing COVID-19. Vox posted an infographic on social media that broke risk of developing COVID-19 down into four categories, lowest risk (home alone or with housemates), moderate risk (outdoor activities), higher risk (outdoor gatherings), and highest risk (indoor gatherings). The infographic includes recommendations about preventive measures that are specific to each category. The infographic clearly conveys how to minimize risk of developing COVID-19 while conveying what preventive measures should be taken regardless of what risk category a person finds themselves in Resnick (2020). Social media messages need to be framed in a way that create an understanding of what activities and behaviors increase risk while promoting what preventive measures are necessary for personal protection based on category of risk.

### Engage Online Influencers and Amplify the Voices of Experts

Espousing opinions and filling social media with uninformed opinions is not productive during an outbreak such as COVID-19. What is needed during outbreaks are facts from trusted organizations such as the World Health Organization, Centers for Disease Control, National Institute of Health or other organizations. Sharing verified facts about COVID-19 reduces fear, anxiety, and increases the adoption of proper protective measures while sharing opinions instills fear and panic. Amplifying the voices of experts increases the number of people that receive accurate and up to date information about COVID-19.

One way to do this is to engage online influencers or celebrities with large social media followings. The World Health Organization has 751,000 followers on Instagram while the Centers for Disease Control has 852,000. In comparison, several celebrities have hundreds of millions of followers. Celebrities can amplify the voices of experts by using their platforms to share messages crafted by experts and health organizations. The “pass mic” imitative began on May 21st, 2020 and allows a celebrity to hand over their Instagram account to medical experts or frontline workers. On May 21st Julia Roberts, who has 8.8 million followers on Instagram, handed her account over to Dr. Anthony Fauci, a leading member of the White House Coronavirus task force. Roberts briefly interviewed Dr. Fauci on YouTube in addition to posting six COVID-19 related posts crafted by Dr. Fauci. Several other celebrities have committed to doing the same (Paisley, 2020). In Vietnam singer Khac Hun partnered with Vietnam’s National Institute to write and promote a song called “ghen co vy” (coronavirus in Vietnamese) to promote hand washing (BBC News, 2020a). The song was turned into a TikTok video choreographed by dancer Quang Dang and has reached millions of users. The video even gained attention and has been promoted by UNICEF (UN News, 2020).

### Craft Messages for Lay Audiences

Often during outbreaks such as COVID-19, those in charge of relaying information to the public use confusing scientific terms or jargon. Organizations such as the World Health Organization (World Health Organization, 2020a), the Centers for Disease Control (Centers for Disease Control and Prevention, 2019), and the American Psychological Association (Lu, 2015) recommend crafting simple and clear messaging. During times of stress and panic it can be difficult for people to process information the way they normally do. Confusing or text heavy social media posts can easily overwhelm people and have an adverse psychological impact. Developing social media messages that are short and to the point increases the chances that people will retain the message. Visuals and infographics can be particularly useful tools for social media (Centers for Disease Control and Prevention, 2019; World Health Organization, 2020a). Several organizations utilize infographics to promote protective measures such as hand washing, social distancing, cleaning and disinfecting objects and surfaces on a regular basis in addition to others. The World Health Organization released a set of infographics, one of which is “COVID-19 – Know the Facts.” The infographic is separated into three sections, one that highlights that COVID-19 is primarily transmitted from person to person, another that outlines other modes of transmission (elevator buttons, doorknobs, pens, etc.), and finally details six ways to reduce the risk of developing COVID-19. In a straightforward and engaging way, this infographic outlines the primary way COVID-19 is spread, other ways it can be transmitted that people are less conscious of, and ways to reduce risk (World Health Organization, 2020a).

### Create Interactive Forums Where the Public Can Access Up-to-Date Information

While crafting accurate and effective social media messages is crucial, messages often contain only information on one aspect of COVID-19. Messages that contain too much text or too much information can become overwhelming (Social Science in Humanitarian Action Platform, 2020). During uncertain and unprecedented times such as COVID-19, people have lots of questions and often few reliable forums in which to ask them. Interactive social media forums that the public can engage with and navigate to find answers to their questions about COVID-19 increases the amount of reliable information that they consume. Interactive platforms also offer a more tailored experience that allows the user to dictate what information they access. Facebook, in collaboration with the World Health Organization, developed an interactive health alert service through Facebook Messenger where users can type in their own question or choose from a dashboard of topics. These dashboard topics include latest numbers, personal protection, a myth of fact quiz, frequently asked questions, travel advice, news and press, and a share option. In a little more than a month, the World Health Organization’s Health Alert System has already reached over 12 million people (Posetti and Matthews, 2018; World Health Organization, 2020b). Interactive platforms such as the Health Alert System offer an opportunity to widen the reach of accurate and up-to-date health information to millions of people worldwide.

### Be Honest About What Is Known and Unknown

Information cannot be withheld from the public out of fear about how they will respond. Trust with the public is built over time, and consistent messages that acknowledge what is known and what is unknown not only helps builds trust it provides health organizations an opportunity to emphasize what preventive measures are known to work and should be adopted (BBC News, 2020b; Ethical Journalism Network, 2020; Social Science in Humanitarian Action Platform, 2020). The absence of communication from trusted sources about COVID-19 also creates an information vacuum that leads to speculation (Lu, 2015). Speculation leads to anxiety, panic, and forces people to formulate their own opinions about what is going on and how to protect themselves. Providing consistent updates about social media is an opportunity for trusted organizations to build trust with the public and release information about what is known and not known. In Vietnam, a country that has recorded zero deaths and under 500 cases of COVID-19 and shares a border with China, social media was used by the government and scientific journalists, to disseminate information about the “strange pneumonia” in China in the early days of the pandemic (Klofstad et al., 2019). La et al. (2020) note that raising awareness among the citizens of Vietnam created trust between the government, civil society, and private individuals and reduced panic about COVID-19 (Klofstad et al., 2019).

The Centers for Disease Control developed a set of 16 sets of social media platform specific (Twitter, Facebook, Instagram) infographics that are meant to inform the public about several different issues surrounding COVID-19 (Centers for Disease Control and Prevention, 2020). The first infographic in the “stop the spread” set begins with “much is unknown about how the virus that causes COVID-19 spread” and goes on to promote preventive measures such as hand washing, disinfecting objects and surfaces in addition to others. These infographics acknowledge that there is risk but highlight that there are known ways preventive measures that will reduce the risk of developing COVID-19.

### Media and Information Literacy

Being responsible consumers of information on social media is important under any circumstances but is even more important during times of crises. The concept of media and information literacy is grounded in the idea that we need to critically assess information that we encounter (BBC News, 2020a). Questioning whether or not a source is credible, cross-checking facts with trusted sources of information about COVID-19, and not falling prey to headlines that are meant to elicit an emotional response are all important (Social Science in Humanitarian Action Platform, 2020). During the COVID-19 pandemic, fake accounts have been created that claim to be academic institutions that are typically reliable sources of information. Recently, an account claiming to be a researcher at Stanford University Hospital posted false claims such as “taking a few sips of water every 15 min” can prevent COVID-19 (Ziv and Wineburg, 2020).

Understanding how to spot misinformation is important in order to curtail misinformation on social media. The “sanitize before you share” campaign created by the News Literacy Project lays out four steps to spot and stop the spread of misinformation on social media by enhancing social media user’s ability to detect it. Before a person shares a post on social media they should pause and not share the post because of their emotional response to it. Next, look through the comments to see if someone has posted a response with a fact check. If there are no fact check responses, do a quick google search to see if the information in the post is supported by trusted health organizations. Finally, if there is no evidence that supports the post, ask for the user’s source. Comments asking for the source show up in the comments section of the post and can also alert other users to the fact that the information in the post is questionable and should be treated with skepticism (News Literacy Project, 2020).

### Use Recommended Hashtags in Posts

Hashtags are used in social media before a relevant keyword or phrase and creates a hyperlink making information about that topic easier to find and engage with. The Centers for Disease Control recommends using #COVID19 whenever posting something COVID-19 related. Hashtags provide a space where people can have open communication about COVID-19 and disseminate information. Other hashtags such as #CoronaVirusFacts are used by trusted organizations or experts to debunk myths about COVID-19. #KnowCOVID is another hashtag that is used that provides people with links to trusted sources of information and shares posts from reliable sources with updates about COVID-19. Others such as #StayHomeStaySafe and #StayHome are used to promote preventive measures. Hashtags have been used in a number of different ways in the pandemic and can be a useful tool for fighting misinformation and promoting preventive measures.

## Limitations

Despite drawing on recommendations and strengths of social media strategies and campaigns from trusted organizations and lessons learned from past epidemics, this article has several limitations. Because of the number of trusted health and communications organizations that have disseminated information about COVID-19 on social media, we were unable to review many recommendations that could inform effective social media campaigns. However, because the best practices we set forth were based on recommendations that were found across organizations and drew upon lessons learned during past epidemics, we are confident that these best practices will improve the quality of social media messaging during COVID-19. Additionally, social media platforms are constantly evolving, and platforms such as TikTok were not popular or were only available in a few countries. While TikTok videos and other campaigns on other social media platforms reach millions of users, whether they increase preventive measures and/or curtail misinformation has not been evaluated. Despite the limitations of this study, we believe our recommendations can serve as a foundation for additional research into increasing the adoption of preventive measures and slowing the spread of misinformation about COVID-19 on social media.

## Conclusion

Developing a set of best practices for crafting social media messages during COVID-19 and expanding their reach has the potential to improve the quality of information on social media. Information about personal protective measures must come from trusted health organizations or experts that have the most up to date information. Misinformation and mixed messages force people to develop their own opinion about the most effective personal protective measures. Misinformation also creates uncertainty about the nature of COVID-19 which leads to increased fear, anxiety, and panic that can be reduced by receiving consistent, straight forward updates and messages from trusted health professionals. Social media is currently riddled with misinformation about COVID-19. While we cannot do anything to eliminate misinformation on social media, these best practices can serve as a foundation for developing effective social media messages, widen the reach of posts by health organizations, and enhance social media user’s ability to detect and share misinformation. Preventive measures are not only important to adopt to protect individuals, but failure to adopt proper preventive measures decreases our ability to control COVID-19.

## Author Contributions

MH wrote the manuscript. SS provided feedback. Both authors contributed to the article and approved the submitted version.

## Conflict of Interest

The authors declare that the research was conducted in the absence of any commercial or financial relationships that could be construed as a potential conflict of interest.
